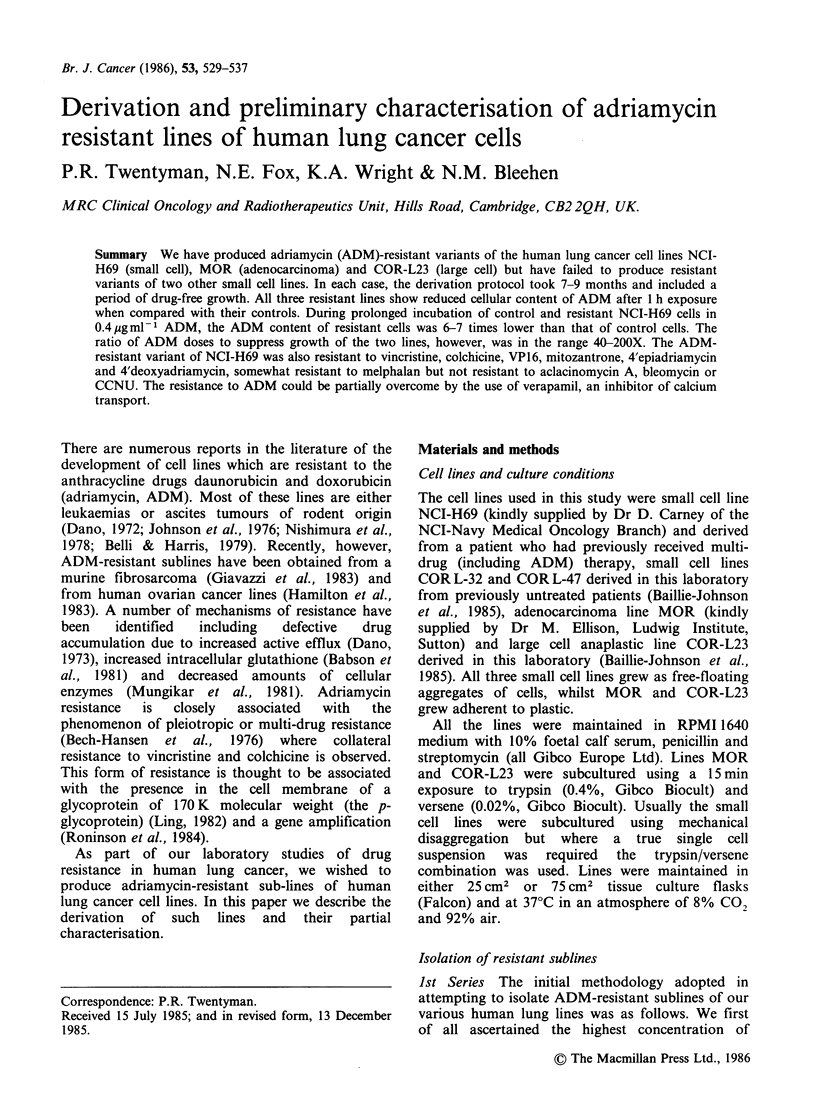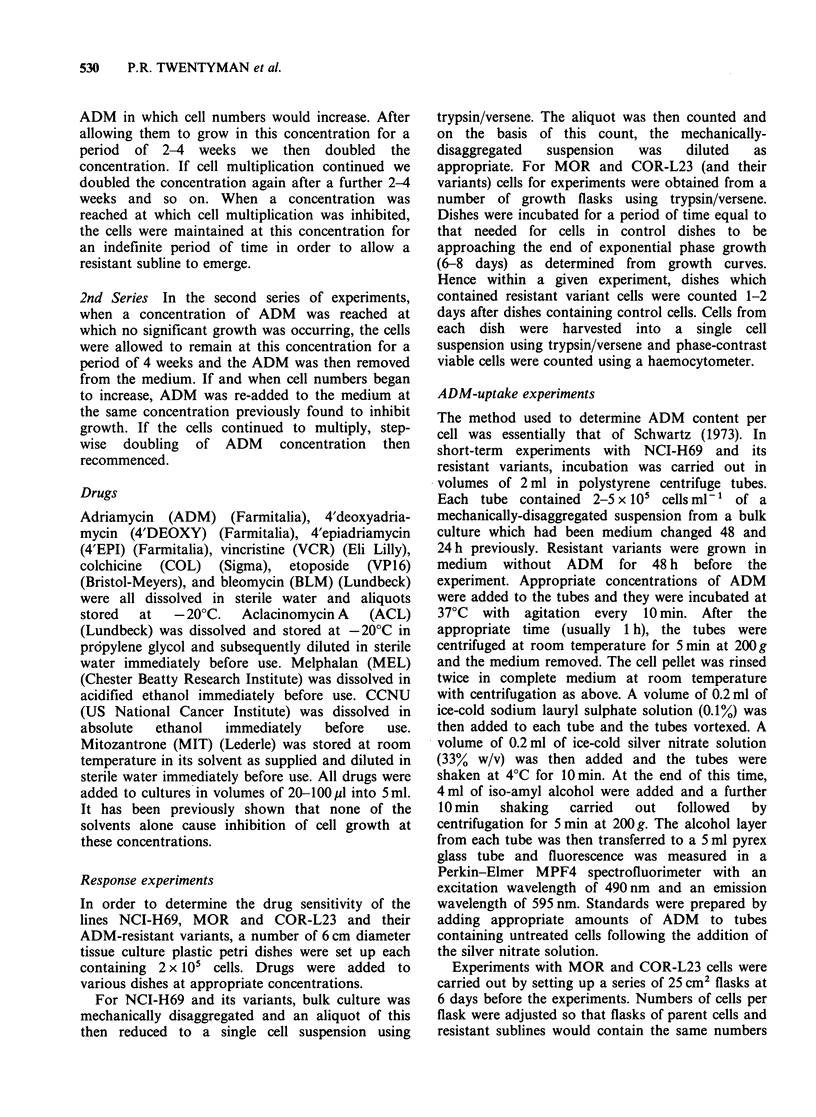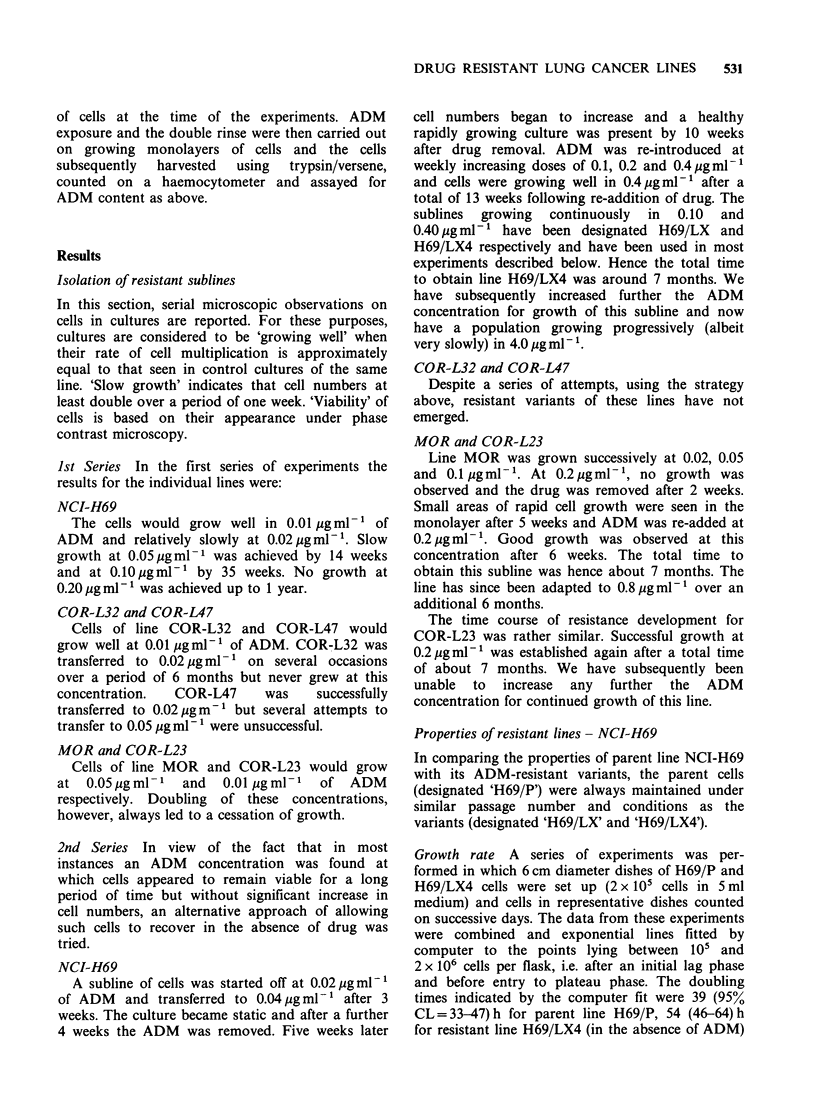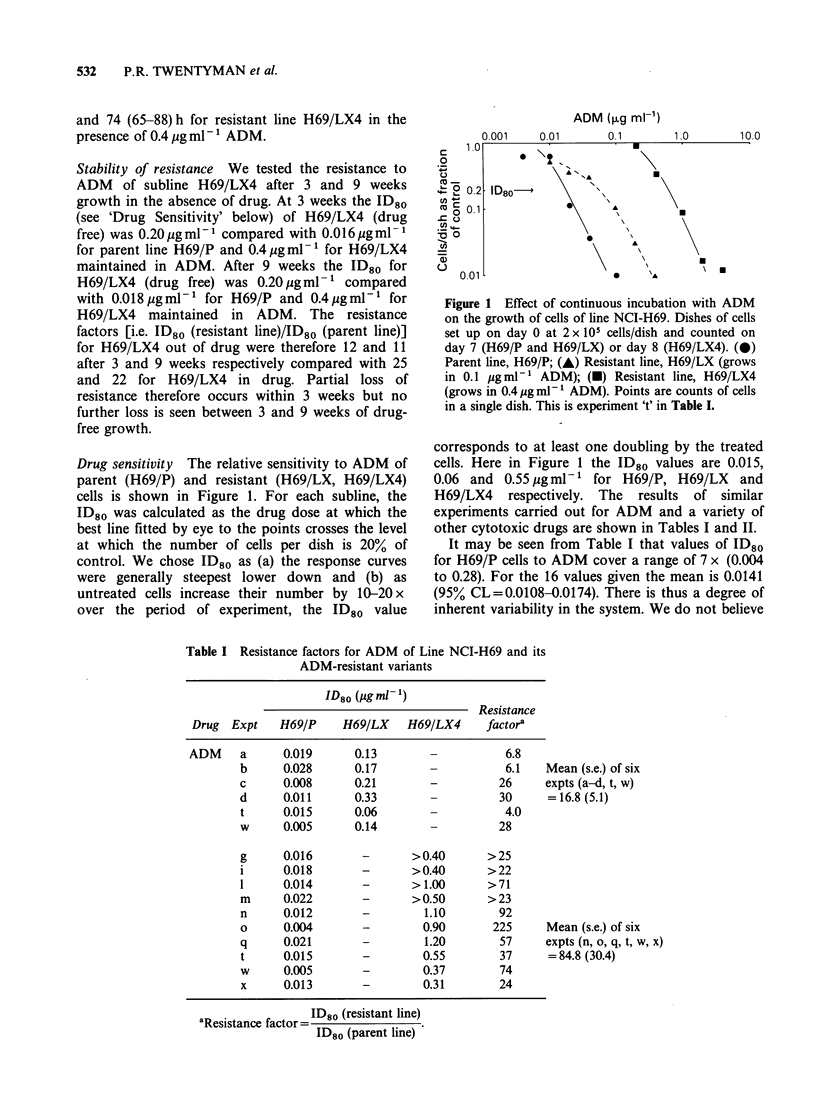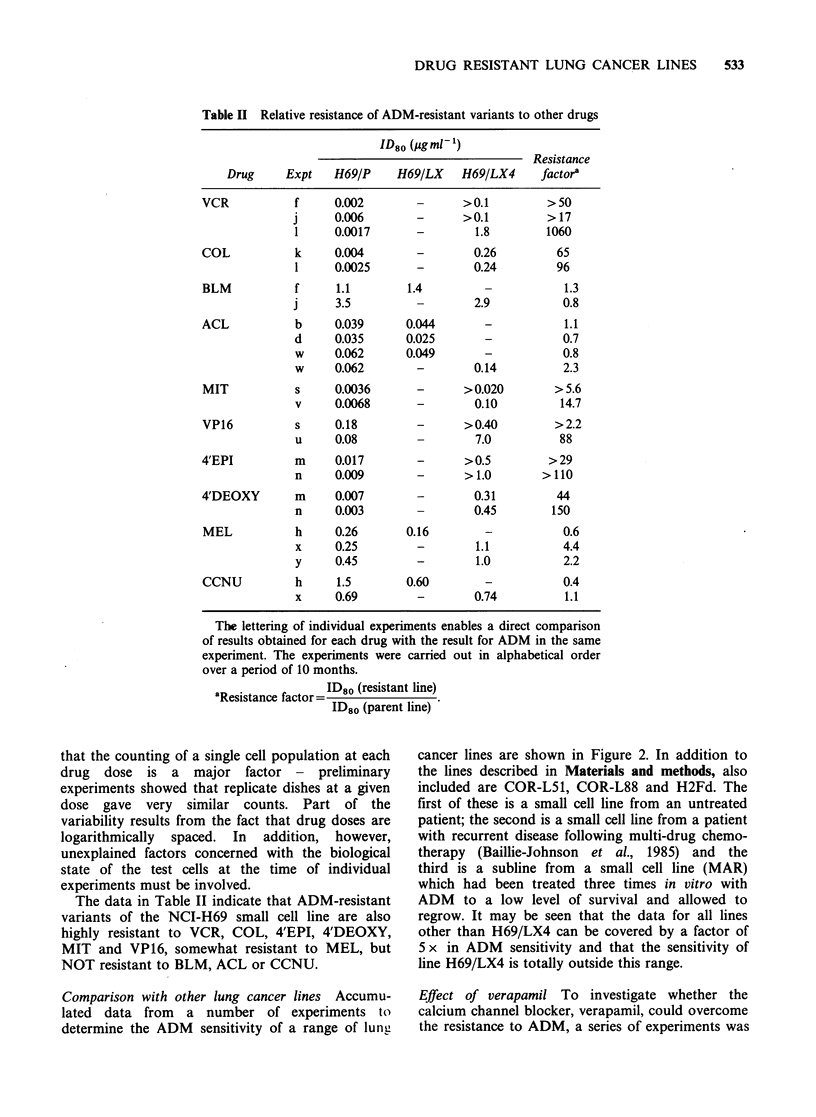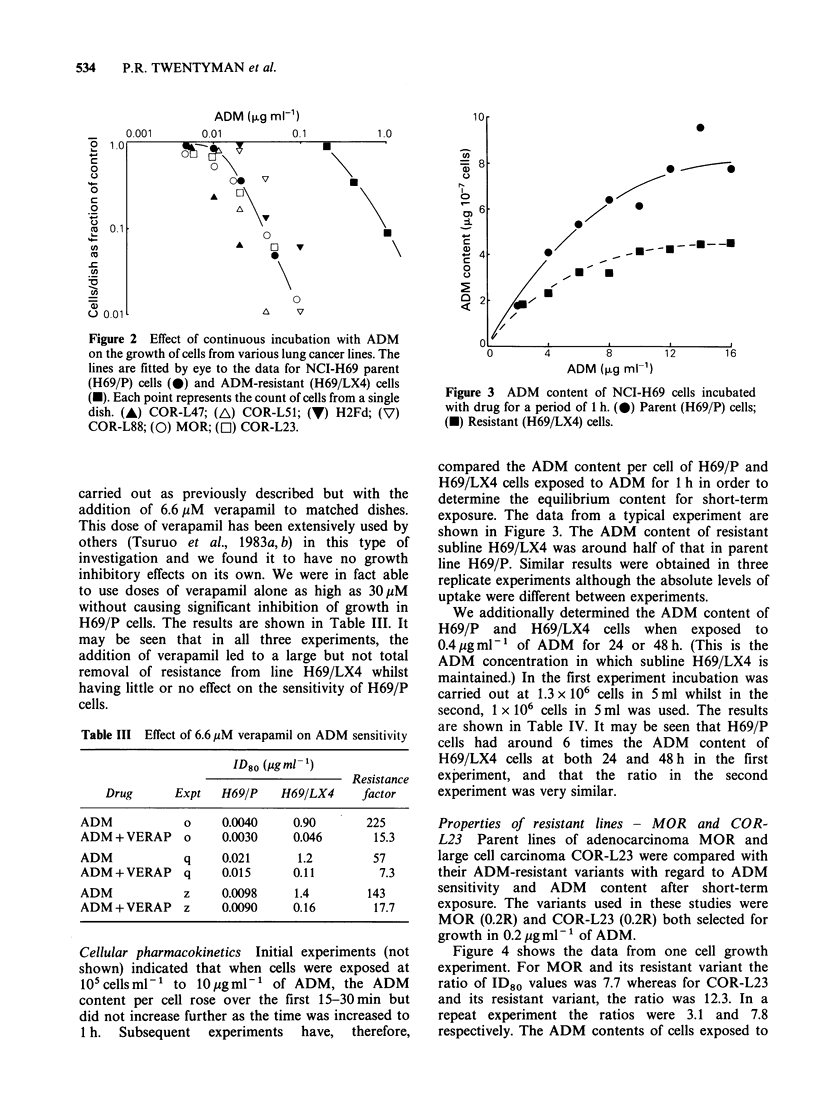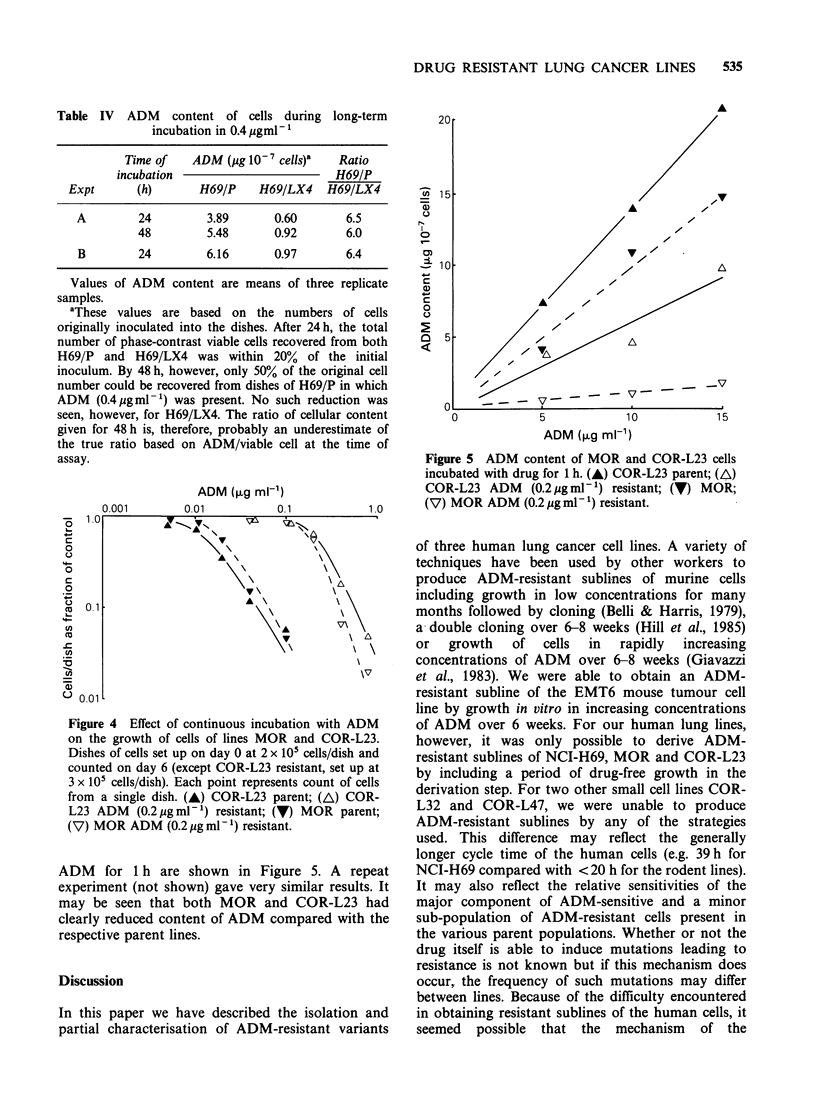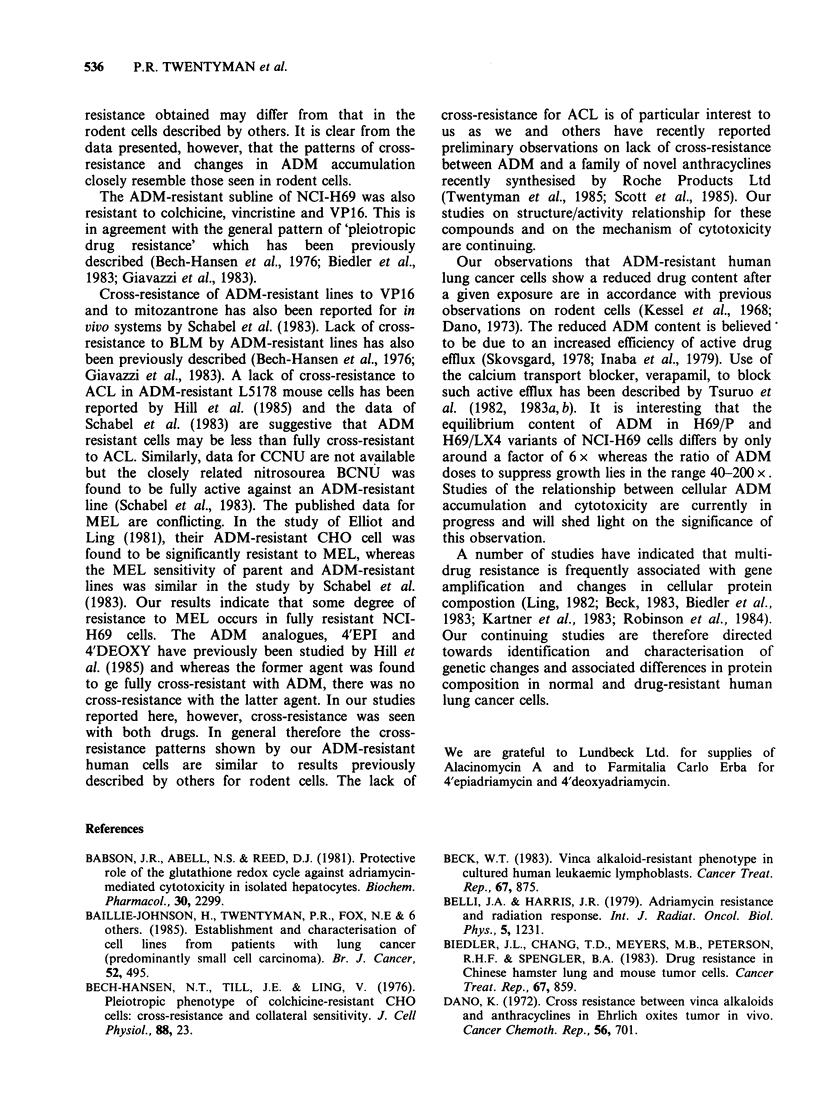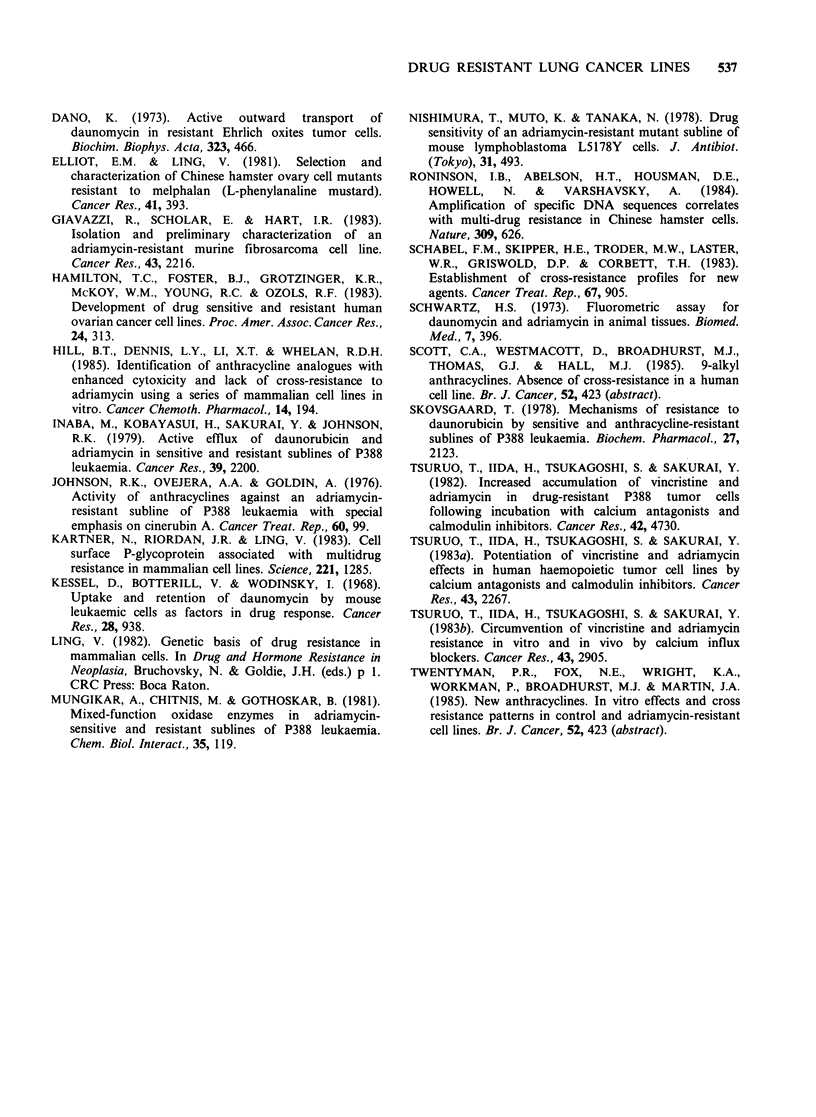# Derivation and preliminary characterisation of adriamycin resistant lines of human lung cancer cells.

**DOI:** 10.1038/bjc.1986.83

**Published:** 1986-04

**Authors:** P. R. Twentyman, N. E. Fox, K. A. Wright, N. M. Bleehen

## Abstract

We have produced adriamycin (ADM)-resistant variants of the human lung cancer cell lines NCI-H69 (small cell), MOR (adenocarcinoma) and COR-L23 (large cell) but have failed to produce resistant variants of two other small cell lines. In each case, the derivation protocol took 7-9 months and included a period of drug-free growth. All three resistant lines show reduced cellular content of ADM after 1 h exposure when compared with their controls. During prolonged incubation of control and resistant NCI-H69 cells in 0.4 microgram ml-1 ADM, the ADM content of resistant cells was 6-7 times lower than that of control cells. The ratio of ADM doses to suppress growth of the two lines, however, was in the range of 40-200X. The ADM-resistant variant of NCI-H69 was also resistant to vincristine, colchicine, VP16, mitozantrone, 4' epiadriamycin and 4' deoxyadriamycin, somewhat resistant to melphalan but not resistant to aclacinomycin A, bleomycin of CCNU. The resistance to ADM could be partially overcome by the use of verapamil, an inhibitor of calcium transport.


*Br. J. Cancer* (1986), **53**, 529–537

### Derivation and preliminary characterisation of adriamycin resistant lines of human lung cancer cells

P.R. Twentyman, N.E. Fox, K.A. Wright & N.M. Bleehen

*MRC Clinical Oncology and Radiotherapeutics Unit, Hills Road, Cambridge, CB2 2QH, UK.*

**Summary** We have produced adriamycin (ADM)-resistant variants of the human lung cancer cell lines NCI-H69 (small cell), MOR (adenocarcinoma) and COR-L23 (large cell) but have failed to produce resistant variants of two other small cell lines. In each case, the derivation protocol took 7–9 months and included a period of drug-free growth. All three resistant lines show reduced cellular content of ADM after 1 h exposure when compared with their controls. During prolonged incubation of control and resistant NCI-H69 cells in $0.4\,\mu g\,ml^{-1}$ ADM, the ADM content of resistant cells was 6–7 times lower than that of control cells. The ratio of ADM doses to suppress growth of the two lines, however, was in the range 40–200X. The ADM-resistant variant of NCI-H69 was also resistant to vincristine, colchicine, VP16, mitozantrone, 4′epiadriamycin and 4′deoxyadriamycin, somewhat resistant to melphalan but not resistant to aclacinomycin A, bleomycin or CCNU. The resistance to ADM could be partially overcome by the use of verapamil, an inhibitor of calcium transport.

There are numerous reports in the literature of the development of cell lines which are resistant to the anthracycline drugs daunorubicin and doxorubicin (adriamycin, ADM). Most of these lines are either leukaemias or ascites tumours of rodent origin (Dano, 1972; Johnson *et al.*, 1976; Nishimura *et al.*, 1978; Belli & Harris, 1979). Recently, however, ADM-resistant sublines have been obtained from a murine fibrosarcoma (Giavazzi *et al.*, 1983) and from human ovarian cancer lines (Hamilton *et al.*, 1983). A number of mechanisms of resistance have been identified including defective drug accumulation due to increased active efflux (Dano, 1973), increased intracellular glutathione (Babson *et al.*, 1981) and decreased amounts of cellular enzymes (Mungikar *et al.*, 1981). Adriamycin resistance is closely associated with the phenomenon of pleiotropic or multi-drug resistance (Bech-Hansen *et al.*, 1976) where collateral resistance to vincristine and colchicine is observed. This form of resistance is thought to be associated with the presence in the cell membrane of a glycoprotein of 170 K molecular weight (the *p*-glycoprotein) (Ling, 1982) and a gene amplification (Roninson *et al.*, 1984).

As part of our laboratory studies of drug resistance in human lung cancer, we wished to produce adriamycin-resistant sub-lines of human lung cancer cell lines. In this paper we describe the derivation of such lines and their partial characterisation.

Correspondence: P.R. Twentyman.
Received 15 July 1985; and in revised form, 13 December 1985.

### Materials and methods

### *Cell lines and culture conditions*

The cell lines used in this study were small cell line NCI-H69 (kindly supplied by Dr D. Carney of the NCI-Navy Medical Oncology Branch) and derived from a patient who had previously received multi-drug (including ADM) therapy, small cell lines COR L-32 and COR L-47 derived in this laboratory from previously untreated patients (Baillie-Johnson *et al.*, 1985), adenocarcinoma line MOR (kindly supplied by Dr M. Ellison, Ludwig Institute, Sutton) and large cell anaplastic line COR-L23 derived in this laboratory (Baillie-Johnson *et al.*, 1985). All three small cell lines grew as free-floating aggregates of cells, whilst MOR and COR-L23 grew adherent to plastic.

All the lines were maintained in RPMI 1640 medium with 10% foetal calf serum, penicillin and streptomycin (all Gibco Europe Ltd). Lines MOR and COR-L23 were subcultured using a 15 min exposure to trypsin (0.4%, Gibco Biocult) and versene (0.02%, Gibco Biocult). Usually the small cell lines were subcultured using mechanical disaggregation but where a true single cell suspension was required the trypsin/versene combination was used. Lines were maintained in either $25\,cm^2$ or $75\,cm^2$ tissue culture flasks (Falcon) and at 37°C in an atmosphere of 8% $CO_2$ and 92% air.

### *Isolation of resistant sublines*

*1st Series* The initial methodology adopted in attempting to isolate ADM-resistant sublines of our various human lung lines was as follows. We first of all ascertained the highest concentration of

© The Macmillan Press Ltd., 1986

ADM in which cell numbers would increase. After allowing them to grow in this concentration for a period of 2–4 weeks we then doubled the concentration. If cell multiplication continued we doubled the concentration again after a further 2–4 weeks and so on. When a concentration was reached at which cell multiplication was inhibited, the cells were maintained at this concentration for an indefinite period of time in order to allow a resistant subline to emerge.

*2nd Series* In the second series of experiments, when a concentration of ADM was reached at which no significant growth was occurring, the cells were allowed to remain at this concentration for a period of 4 weeks and the ADM was then removed from the medium. If and when cell numbers began to increase, ADM was re-added to the medium at the same concentration previously found to inhibit growth. If the cells continued to multiply, step-wise doubling of ADM concentration then recommenced.

*Drugs*

Adriamycin (ADM) (Farmitalia), 4′deoxyadriamycin (4′DEOXY) (Farmitalia), 4′epiadriamycin (4′EPI) (Farmitalia), vincristine (VCR) (Eli Lilly), colchicine (COL) (Sigma), etoposide (VP16) (Bristol-Meyers), and bleomycin (BLM) (Lundbeck) were all dissolved in sterile water and aliquots stored at −20°C. Aclacinomycin A (ACL) (Lundbeck) was dissolved and stored at −20°C in propylene glycol and subsequently diluted in sterile water immediately before use. Melphalan (MEL) (Chester Beatty Research Institute) was dissolved in acidified ethanol immediately before use. CCNU (US National Cancer Institute) was dissolved in absolute ethanol immediately before use. Mitozantrone (MIT) (Lederle) was stored at room temperature in its solvent as supplied and diluted in sterile water immediately before use. All drugs were added to cultures in volumes of 20–100 $\mu$l into 5 ml. It has been previously shown that none of the solvents alone cause inhibition of cell growth at these concentrations.

*Response experiments*

In order to determine the drug sensitivity of the lines NCI-H69, MOR and COR-L23 and their ADM-resistant variants, a number of 6 cm diameter tissue culture plastic petri dishes were set up each containing $2 \times 10^5$ cells. Drugs were added to various dishes at appropriate concentrations.

For NCI-H69 and its variants, bulk culture was mechanically disaggregated and an aliquot of this then reduced to a single cell suspension using trypsin/versene. The aliquot was then counted and on the basis of this count, the mechanically-disaggregated suspension was diluted as appropriate. For MOR and COR-L23 (and their variants) cells for experiments were obtained from a number of growth flasks using trypsin/versene. Dishes were incubated for a period of time equal to that needed for cells in control dishes to be approaching the end of exponential phase growth (6–8 days) as determined from growth curves. Hence within a given experiment, dishes which contained resistant variant cells were counted 1–2 days after dishes containing control cells. Cells from each dish were harvested into a single cell suspension using trypsin/versene and phase-contrast viable cells were counted using a haemocytometer.

*ADM-uptake experiments*

The method used to determine ADM content per cell was essentially that of Schwartz (1973). In short-term experiments with NCI-H69 and its resistant variants, incubation was carried out in volumes of 2 ml in polystyrene centrifuge tubes. Each tube contained $2–5 \times 10^5$ cells ml$^{-1}$ of a mechanically-disaggregated suspension from a bulk culture which had been medium changed 48 and 24 h previously. Resistant variants were grown in medium without ADM for 48 h before the experiment. Appropriate concentrations of ADM were added to the tubes and they were incubated at 37°C with agitation every 10 min. After the appropriate time (usually 1 h), the tubes were centrifuged at room temperature for 5 min at 200 *g* and the medium removed. The cell pellet was rinsed twice in complete medium at room temperature with centrifugation as above. A volume of 0.2 ml of ice-cold sodium lauryl sulphate solution (0.1%) was then added to each tube and the tubes vortexed. A volume of 0.2 ml of ice-cold silver nitrate solution (33% w/v) was then added and the tubes were shaken at 4°C for 10 min. At the end of this time, 4 ml of iso-amyl alcohol were added and a further 10 min shaking carried out followed by centrifugation for 5 min at 200 *g*. The alcohol layer from each tube was then transferred to a 5 ml pyrex glass tube and fluorescence was measured in a Perkin–Elmer MPF4 spectrofluorimeter with an excitation wavelength of 490 nm and an emission wavelength of 595 nm. Standards were prepared by adding appropriate amounts of ADM to tubes containing untreated cells following the addition of the silver nitrate solution.

Experiments with MOR and COR-L23 cells were carried out by setting up a series of 25 cm$^2$ flasks at 6 days before the experiments. Numbers of cells per flask were adjusted so that flasks of parent cells and resistant sublines would contain the same numbers of cells at the time of the experiments. ADM exposure and the double rinse were then carried out on growing monolayers of cells and the cells subsequently harvested using trypsin/versene, counted on a haemocytometer and assayed for ADM content as above.

### Results

*Isolation of resistant sublines*

In this section, serial microscopic observations on cells in cultures are reported. For these purposes, cultures are considered to be 'growing well' when their rate of cell multiplication is approximately equal to that seen in control cultures of the same line. 'Slow growth' indicates that cell numbers at least double over a period of one week. 'Viability' of cells is based on their appearance under phase contrast microscopy.

*1st Series* In the first series of experiments the results for the individual lines were:

*NCI-H69*

The cells would grow well in 0.01 $\mu g\,ml^{-1}$ of ADM and relatively slowly at 0.02 $\mu g\,ml^{-1}$. Slow growth at 0.05 $\mu g\,ml^{-1}$ was achieved by 14 weeks and at 0.10 $\mu g\,ml^{-1}$ by 35 weeks. No growth at 0.20 $\mu g\,ml^{-1}$ was achieved up to 1 year.

*COR-L32 and COR-L47*

Cells of line COR-L32 and COR-L47 would grow well at 0.01 $\mu g\,ml^{-1}$ of ADM. COR-L32 was transferred to 0.02 $\mu g\,ml^{-1}$ on several occasions over a period of 6 months but never grew at this concentration. COR-L47 was successfully transferred to 0.02 $\mu g\,m^{-1}$ but several attempts to transfer to 0.05 $\mu g\,ml^{-1}$ were unsuccessful.

*MOR and COR-L23*

Cells of line MOR and COR-L23 would grow at 0.05 $\mu g\,ml^{-1}$ and 0.01 $\mu g\,ml^{-1}$ of ADM respectively. Doubling of these concentrations, however, always led to a cessation of growth.

*2nd Series* In view of the fact that in most instances an ADM concentration was found at which cells appeared to remain viable for a long period of time but without significant increase in cell numbers, an alternative approach of allowing such cells to recover in the absence of drug was tried.

*NCI-H69*

A subline of cells was started off at 0.02 $\mu g\,ml^{-1}$ of ADM and transferred to 0.04 $\mu g\,ml^{-1}$ after 3 weeks. The culture became static and after a further 4 weeks the ADM was removed. Five weeks later cell numbers began to increase and a healthy rapidly growing culture was present by 10 weeks after drug removal. ADM was re-introduced at weekly increasing doses of 0.1, 0.2 and 0.4 $\mu g\,ml^{-1}$ and cells were growing well in 0.4 $\mu g\,ml^{-1}$ after a total of 13 weeks following re-addition of drug. The sublines growing continuously in 0.10 and 0.40 $\mu g\,ml^{-1}$ have been designated H69/LX and H69/LX4 respectively and have been used in most experiments described below. Hence the total time to obtain line H69/LX4 was around 7 months. We have subsequently increased further the ADM concentration for growth of this subline and now have a population growing progressively (albeit very slowly) in 4.0 $\mu g\,ml^{-1}$.

*COR-L32 and COR-L47*

Despite a series of attempts, using the strategy above, resistant variants of these lines have not emerged.

*MOR and COR-L23*

Line MOR was grown successively at 0.02, 0.05 and 0.1 $\mu g\,ml^{-1}$. At 0.2 $\mu g\,ml^{-1}$, no growth was observed and the drug was removed after 2 weeks. Small areas of rapid cell growth were seen in the monolayer after 5 weeks and ADM was re-added at 0.2 $\mu g\,ml^{-1}$. Good growth was observed at this concentration after 6 weeks. The total time to obtain this subline was hence about 7 months. The line has since been adapted to 0.8 $\mu g\,ml^{-1}$ over an additional 6 months.

The time course of resistance development for COR-L23 was rather similar. Successful growth at 0.2 $\mu g\,ml^{-1}$ was established again after a total time of about 7 months. We have subsequently been unable to increase any further the ADM concentration for continued growth of this line.

*Properties of resistant lines – NCI-H69*

In comparing the properties of parent line NCI-H69 with its ADM-resistant variants, the parent cells (designated 'H69/P') were always maintained under similar passage number and conditions as the variants (designated 'H69/LX' and 'H69/LX4').

*Growth rate* A series of experiments was performed in which 6 cm diameter dishes of H69/P and H69/LX4 cells were set up ($2\times10^5$ cells in 5 ml medium) and cells in representative dishes counted on successive days. The data from these experiments were combined and exponential lines fitted by computer to the points lying between $10^5$ and $2\times10^6$ cells per flask, i.e. after an initial lag phase and before entry to plateau phase. The doubling times indicated by the computer fit were 39 (95% CL = 33–47) h for parent line H69/P, 54 (46–64) h for resistant line H69/LX4 (in the absence of ADM)

and 74 (65–88) h for resistant line H69/LX4 in the presence of 0.4 $\mu$g ml$^{-1}$ ADM.

*Stability of resistance* We tested the resistance to ADM of subline H69/LX4 after 3 and 9 weeks growth in the absence of drug. At 3 weeks the $ID_{80}$ (see 'Drug Sensitivity' below) of H69/LX4 (drug free) was 0.20 $\mu$g ml$^{-1}$ compared with 0.016 $\mu$g ml$^{-1}$ for parent line H69/P and 0.4 $\mu$g ml$^{-1}$ for H69/LX4 maintained in ADM. After 9 weeks the $ID_{80}$ for H69/LX4 (drug free) was 0.20 $\mu$g ml$^{-1}$ compared with 0.018 $\mu$g ml$^{-1}$ for H69/P and 0.4 $\mu$g ml$^{-1}$ for H69/LX4 maintained in ADM. The resistance factors [i.e. $ID_{80}$ (resistant line)/$ID_{80}$ (parent line)] for H69/LX4 out of drug were therefore 12 and 11 after 3 and 9 weeks respectively compared with 25 and 22 for H69/LX4 in drug. Partial loss of resistance therefore occurs within 3 weeks but no further loss is seen between 3 and 9 weeks of drug-free growth.

*Drug sensitivity* The relative sensitivity to ADM of parent (H69/P) and resistant (H69/LX, H69/LX4) cells is shown in Figure 1. For each subline, the $ID_{80}$ was calculated as the drug dose at which the best line fitted by eye to the points crosses the level at which the number of cells per dish is 20% of control. We chose $ID_{80}$ as (a) the response curves were generally steepest lower down and (b) as untreated cells increase their number by 10–20 × over the period of experiment, the $ID_{80}$ value

**Figure 1** Effect of continuous incubation with ADM on the growth of cells of line NCI-H69. Dishes of cells set up on day 0 at $2 \times 10^5$ cells/dish and counted on day 7 (H69/P and H69/LX) or day 8 (H69/LX4). (●) Parent line, H69/P; (▲) Resistant line, H69/LX (grows in 0.1 $\mu$g ml$^{-1}$ ADM); (■) Resistant line, H69/LX4 (grows in 0.4 $\mu$g ml$^{-1}$ ADM). Points are counts of cells in a single dish. This is experiment 't' in **Table I.**

corresponds to at least one doubling by the treated cells. Here in Figure 1 the $ID_{80}$ values are 0.015, 0.06 and 0.55 $\mu$g ml$^{-1}$ for H69/P, H69/LX and H69/LX4 respectively. The results of similar experiments carried out for ADM and a variety of other cytotoxic drugs are shown in Tables I and II.

It may be seen from Table I that values of $ID_{80}$ for H69/P cells to ADM cover a range of 7 × (0.004 to 0.28). For the 16 values given the mean is 0.0141 (95% CL = 0.0108–0.0174). There is thus a degree of inherent variability in the system. We do not believe

**Table I** Resistance factors for ADM of Line NCI-H69 and its ADM-resistant variants

| | | *$ID_{80}$ ($\mu$g ml$^{-1}$)* | | | | |
|---|---|---|---|---|---|---|
| *Drug* | *Expt* | *H69/P* | *H69/LX* | *H69/LX4* | *Resistance factor*[a] | |
| ADM | a | 0.019 | 0.13 | – | 6.8 | |
| | b | 0.028 | 0.17 | – | 6.1 | Mean (s.e.) of six |
| | c | 0.008 | 0.21 | – | 26 | expts (a–d, t, w) |
| | d | 0.011 | 0.33 | – | 30 | = 16.8 (5.1) |
| | t | 0.015 | 0.06 | – | 4.0 | |
| | w | 0.005 | 0.14 | – | 28 | |
| | g | 0.016 | – | >0.40 | >25 | |
| | i | 0.018 | – | >0.40 | >22 | |
| | l | 0.014 | – | >1.00 | >71 | |
| | m | 0.022 | – | >0.50 | >23 | |
| | n | 0.012 | – | 1.10 | 92 | |
| | o | 0.004 | – | 0.90 | 225 | Mean (s.e.) of six |
| | q | 0.021 | – | 1.20 | 57 | expts (n, o, q, t, w, x) |
| | t | 0.015 | – | 0.55 | 37 | = 84.8 (30.4) |
| | w | 0.005 | – | 0.37 | 74 | |
| | x | 0.013 | – | 0.31 | 24 | |

[a]Resistance factor = $\frac{ID_{80} \text{ (resistant line)}}{ID_{80} \text{ (parent line)}}$.

**Table II** Relative resistance of ADM-resistant variants to other drugs

| *Drug* | *Expt* | *$ID_{80}$ ($\mu g\, ml^{-1}$)* *H69/P* | *H69/LX* | *H69/LX4* | *Resistance factor*[a] |
|---|---|---|---|---|---|
| VCR | f | 0.002 | – | >0.1 | >50 |
| | j | 0.006 | – | >0.1 | >17 |
| | l | 0.0017 | – | 1.8 | 1060 |
| COL | k | 0.004 | – | 0.26 | 65 |
| | l | 0.0025 | – | 0.24 | 96 |
| BLM | f | 1.1 | 1.4 | – | 1.3 |
| | j | 3.5 | – | 2.9 | 0.8 |
| ACL | b | 0.039 | 0.044 | – | 1.1 |
| | d | 0.035 | 0.025 | – | 0.7 |
| | w | 0.062 | 0.049 | – | 0.8 |
| | w | 0.062 | – | 0.14 | 2.3 |
| MIT | s | 0.0036 | – | >0.020 | >5.6 |
| | v | 0.0068 | – | 0.10 | 14.7 |
| VP16 | s | 0.18 | – | >0.40 | >2.2 |
| | u | 0.08 | – | 7.0 | 88 |
| 4'EPI | m | 0.017 | – | >0.5 | >29 |
| | n | 0.009 | – | >1.0 | >110 |
| 4'DEOXY | m | 0.007 | – | 0.31 | 44 |
| | n | 0.003 | – | 0.45 | 150 |
| MEL | h | 0.26 | 0.16 | – | 0.6 |
| | x | 0.25 | – | 1.1 | 4.4 |
| | y | 0.45 | – | 1.0 | 2.2 |
| CCNU | h | 1.5 | 0.60 | – | 0.4 |
| | x | 0.69 | – | 0.74 | 1.1 |

The lettering of individual experiments enables a direct comparison of results obtained for each drug with the result for ADM in the same experiment. The experiments were carried out in alphabetical order over a period of 10 months.

[a]Resistance factor $= \dfrac{ID_{80}\text{ (resistant line)}}{ID_{80}\text{ (parent line)}}$.

that the counting of a single cell population at each drug dose is a major factor – preliminary experiments showed that replicate dishes at a given dose gave very similar counts. Part of the variability results from the fact that drug doses are logarithmically spaced. In addition, however, unexplained factors concerned with the biological state of the test cells at the time of individual experiments must be involved.

The data in Table II indicate that ADM-resistant variants of the NCI-H69 small cell line are also highly resistant to VCR, COL, 4'EPI, 4'DEOXY, MIT and VP16, somewhat resistant to MEL, but NOT resistant to BLM, ACL or CCNU.

*Comparison with other lung cancer lines* Accumulated data from a number of experiments to determine the ADM sensitivity of a range of lung cancer lines are shown in Figure 2. In addition to the lines described in **Materials and methods,** also included are COR-L51, COR-L88 and H2Fd. The first of these is a small cell line from an untreated patient; the second is a small cell line from a patient with recurrent disease following multi-drug chemotherapy (Baillie-Johnson *et al.*, 1985) and the third is a subline from a small cell line (MAR) which had been treated three times *in vitro* with ADM to a low level of survival and allowed to regrow. It may be seen that the data for all lines other than H69/LX4 can be covered by a factor of 5× in ADM sensitivity and that the sensitivity of line H69/LX4 is totally outside this range.

*Effect of verapamil* To investigate whether the calcium channel blocker, verapamil, could overcome the resistance to ADM, a series of experiments was

**Figure 2** Effect of continuous incubation with ADM on the growth of cells from various lung cancer lines. The lines are fitted by eye to the data for NCI-H69 parent (H69/P) cells (●) and ADM-resistant (H69/LX4) cells (■). Each point represents the count of cells from a single dish. (▲) COR-L47; (△) COR-L51; (▼) H2Fd; (▽) COR-L88; (○) MOR; (□) COR-L23.

**Figure 3** ADM content of NCI-H69 cells incubated with drug for a period of 1 h. (●) Parent (H69/P) cells; (■) Resistant (H69/LX4) cells.

carried out as previously described but with the addition of 6.6 $\mu$M verapamil to matched dishes. This dose of verapamil has been extensively used by others (Tsuruo *et al.*, 1983*a*, *b*) in this type of investigation and we found it to have no growth inhibitory effects on its own. We were in fact able to use doses of verapamil alone as high as 30 $\mu$M without causing significant inhibition of growth in H69/P cells. The results are shown in Table III. It may be seen that in all three experiments, the addition of verapamil led to a large but not total removal of resistance from line H69/LX4 whilst having little or no effect on the sensitivity of H69/P cells.

**Table III** Effect of 6.6 $\mu$M verapamil on ADM sensitivity

| | | $ID_{80}$ ($\mu g\ ml^{-1}$) | | |
|---|---|---|---|---|
| *Drug* | *Expt* | *H69/P* | *H69/LX4* | *Resistance factor* |
| ADM | o | 0.0040 | 0.90 | 225 |
| ADM + VERAP | o | 0.0030 | 0.046 | 15.3 |
| ADM | q | 0.021 | 1.2 | 57 |
| ADM + VERAP | q | 0.015 | 0.11 | 7.3 |
| ADM | z | 0.0098 | 1.4 | 143 |
| ADM + VERAP | z | 0.0090 | 0.16 | 17.7 |

*Cellular pharmacokinetics* Initial experiments (not shown) indicated that when cells were exposed at $10^5$ cells ml$^{-1}$ to 10 $\mu$g ml$^{-1}$ of ADM, the ADM content per cell rose over the first 15–30 min but did not increase further as the time was increased to 1 h. Subsequent experiments have, therefore, compared the ADM content per cell of H69/P and H69/LX4 cells exposed to ADM for 1 h in order to determine the equilibrium content for short-term exposure. The data from a typical experiment are shown in Figure 3. The ADM content of resistant subline H69/LX4 was around half of that in parent line H69/P. Similar results were obtained in three replicate experiments although the absolute levels of uptake were different between experiments.

We additionally determined the ADM content of H69/P and H69/LX4 cells when exposed to 0.4 $\mu$g ml$^{-1}$ of ADM for 24 or 48 h. (This is the ADM concentration in which subline H69/LX4 is maintained.) In the first experiment incubation was carried out at $1.3 \times 10^6$ cells in 5 ml whilst in the second, $1 \times 10^6$ cells in 5 ml was used. The results are shown in Table IV. It may be seen that H69/P cells had around 6 times the ADM content of H69/LX4 cells at both 24 and 48 h in the first experiment, and that the ratio in the second experiment was very similar.

*Properties of resistant lines – MOR and COR-L23* Parent lines of adenocarcinoma MOR and large cell carcinoma COR-L23 were compared with their ADM-resistant variants with regard to ADM sensitivity and ADM content after short-term exposure. The variants used in these studies were MOR (0.2R) and COR-L23 (0.2R) both selected for growth in 0.2 $\mu$g ml$^{-1}$ of ADM.

Figure 4 shows the data from one cell growth experiment. For MOR and its resistant variant the ratio of $ID_{80}$ values was 7.7 whereas for COR-L23 and its resistant variant, the ratio was 12.3. In a repeat experiment the ratios were 3.1 and 7.8 respectively. The ADM contents of cells exposed to

**Table IV** ADM content of cells during long-term incubation in 0.4 $\mu$gml$^{-1}$

| *Expt* | *Time of incubation (h)* | *ADM ($\mu$g $10^{-7}$ cells)*[a] H69/P | H69/LX4 | *Ratio H69/P / H69/LX4* |
|---|---|---|---|---|
| A | 24 | 3.89 | 0.60 | 6.5 |
| | 48 | 5.48 | 0.92 | 6.0 |
| B | 24 | 6.16 | 0.97 | 6.4 |

Values of ADM content are means of three replicate samples.

[a]These values are based on the numbers of cells originally inoculated into the dishes. After 24 h, the total number of phase-contrast viable cells recovered from both H69/P and H69/LX4 was within 20% of the initial inoculum. By 48 h, however, only 50% of the original cell number could be recovered from dishes of H69/P in which ADM (0.4 $\mu$g ml$^{-1}$) was present. No such reduction was seen, however, for H69/LX4. The ratio of cellular content given for 48 h is, therefore, probably an underestimate of the true ratio based on ADM/viable cell at the time of assay.

**Figure 4** Effect of continuous incubation with ADM on the growth of cells of lines MOR and COR-L23. Dishes of cells set up on day 0 at $2 \times 10^5$ cells/dish and counted on day 6 (except COR-L23 resistant, set up at $3 \times 10^5$ cells/dish). Each point represents count of cells from a single dish. (▲) COR-L23 parent; (△) COR-L23 ADM (0.2 $\mu$g ml$^{-1}$) resistant; (▼) MOR parent; (▽) MOR ADM (0.2 $\mu$g ml$^{-1}$) resistant.

ADM for 1 h are shown in Figure 5. A repeat experiment (not shown) gave very similar results. It may be seen that both MOR and COR-L23 had clearly reduced content of ADM compared with the respective parent lines.

### Discussion

In this paper we have described the isolation and partial characterisation of ADM-resistant variants

**Figure 5** ADM content of MOR and COR-L23 cells incubated with drug for 1 h. (▲) COR-L23 parent; (△) COR-L23 ADM (0.2 $\mu$g ml$^{-1}$) resistant; (▼) MOR; (▽) MOR ADM (0.2 $\mu$g ml$^{-1}$) resistant.

of three human lung cancer cell lines. A variety of techniques have been used by other workers to produce ADM-resistant sublines of murine cells including growth in low concentrations for many months followed by cloning (Belli & Harris, 1979), a double cloning over 6–8 weeks (Hill *et al.*, 1985) or growth of cells in rapidly increasing concentrations of ADM over 6–8 weeks (Giavazzi *et al.*, 1983). We were able to obtain an ADM-resistant subline of the EMT6 mouse tumour cell line by growth *in vitro* in increasing concentrations of ADM over 6 weeks. For our human lung lines, however, it was only possible to derive ADM-resistant sublines of NCI-H69, MOR and COR-L23 by including a period of drug-free growth in the derivation step. For two other small cell lines COR-L32 and COR-L47, we were unable to produce ADM-resistant sublines by any of the strategies used. This difference may reflect the generally longer cycle time of the human cells (e.g. 39 h for NCI-H69 compared with <20 h for the rodent lines). It may also reflect the relative sensitivities of the major component of ADM-sensitive and a minor sub-population of ADM-resistant cells present in the various parent populations. Whether or not the drug itself is able to induce mutations leading to resistance is not known but if this mechanism does occur, the frequency of such mutations may differ between lines. Because of the difficulty encountered in obtaining resistant sublines of the human cells, it seemed possible that the mechanism of the resistance obtained may differ from that in the rodent cells described by others. It is clear from the data presented, however, that the patterns of cross-resistance and changes in ADM accumulation closely resemble those seen in rodent cells.

The ADM-resistant subline of NCI-H69 was also resistant to colchicine, vincristine and VP16. This is in agreement with the general pattern of 'pleiotropic drug resistance' which has been previously described (Bech-Hansen *et al.*, 1976; Biedler *et al.*, 1983; Giavazzi *et al.*, 1983).

Cross-resistance of ADM-resistant lines to VP16 and to mitozantrone has also been reported for *in vivo* systems by Schabel *et al.* (1983). Lack of cross-resistance to BLM by ADM-resistant lines has also been previously described (Bech-Hansen *et al.*, 1976; Giavazzi *et al.*, 1983). A lack of cross-resistance to ACL in ADM-resistant L5178 mouse cells has been reported by Hill *et al.* (1985) and the data of Schabel *et al.* (1983) are suggestive that ADM resistant cells may be less than fully cross-resistant to ACL. Similarly, data for CCNU are not available but the closely related nitrosourea BCNU was found to be fully active against an ADM-resistant line (Schabel *et al.*, 1983). The published data for MEL are conflicting. In the study of Elliot and Ling (1981), their ADM-resistant CHO cell was found to be significantly resistant to MEL, whereas the MEL sensitivity of parent and ADM-resistant lines was similar in the study by Schabel *et al.* (1983). Our results indicate that some degree of resistance to MEL occurs in fully resistant NCI-H69 cells. The ADM analogues, 4'EPI and 4'DEOXY have previously been studied by Hill *et al.* (1985) and whereas the former agent was found to ge fully cross-resistant with ADM, there was no cross-resistance with the latter agent. In our studies reported here, however, cross-resistance was seen with both drugs. In general therefore the cross-resistance patterns shown by our ADM-resistant human cells are similar to results previously described by others for rodent cells. The lack of cross-resistance for ACL is of particular interest to us as we and others have recently reported preliminary observations on lack of cross-resistance between ADM and a family of novel anthracyclines recently synthesised by Roche Products Ltd (Twentyman *et al.*, 1985; Scott *et al.*, 1985). Our studies on structure/activity relationship for these compounds and on the mechanism of cytotoxicity are continuing.

Our observations that ADM-resistant human lung cancer cells show a reduced drug content after a given exposure are in accordance with previous observations on rodent cells (Kessel *et al.*, 1968; Dano, 1973). The reduced ADM content is believed to be due to an increased efficiency of active drug efflux (Skovsgard, 1978; Inaba *et al.*, 1979). Use of the calcium transport blocker, verapamil, to block such active efflux has been described by Tsuruo *et al.* (1982, 1983*a*, *b*). It is interesting that the equilibrium content of ADM in H69/P and H69/LX4 variants of NCI-H69 cells differs by only around a factor of 6× whereas the ratio of ADM doses to suppress growth lies in the range 40–200×. Studies of the relationship between cellular ADM accumulation and cytotoxicity are currently in progress and will shed light on the significance of this observation.

A number of studies have indicated that multi-drug resistance is frequently associated with gene amplification and changes in cellular protein compostion (Ling, 1982; Beck, 1983, Biedler *et al.*, 1983; Kartner *et al.*, 1983; Robinson *et al.*, 1984). Our continuing studies are therefore directed towards identification and characterisation of genetic changes and associated differences in protein composition in normal and drug-resistant human lung cancer cells.

We are grateful to Lundbeck Ltd. for supplies of Alacinomycin A and to Farmitalia Carlo Erba for 4'epiadriamycin and 4'deoxyadriamycin.

### References

BABSON, J.R., ABELL, N.S. & REED, D.J. (1981). Protective role of the glutathione redox cycle against adriamycin-mediated cytotoxicity in isolated hepatocytes. *Biochem. Pharmacol.*, **30**, 2299.

BAILLIE-JOHNSON, H., TWENTYMAN, P.R., FOX, N.E & 6 others. (1985). Establishment and characterisation of cell lines from patients with lung cancer (predominantly small cell carcinoma). *Br. J. Cancer*, **52**, 495.

BECH-HANSEN, N.T., TILL, J.E. & LING, V. (1976). Pleiotropic phenotype of colchicine-resistant CHO cells: cross-resistance and collateral sensitivity. *J. Cell Physiol.*, **88**, 23.

BECK, W.T. (1983). Vinca alkaloid-resistant phenotype in cultured human leukaemic lymphoblasts. *Cancer Treat. Rep.*, **67**, 875.

BELLI, J.A. & HARRIS, J.R. (1979). Adriamycin resistance and radiation response. *Int. J. Radiat. Oncol. Biol. Phys.*, **5**, 1231.

BIEDLER, J.L., CHANG, T.D., MEYERS, M.B., PETERSON, R.H.F. & SPENGLER, B.A. (1983). Drug resistance in Chinese hamster lung and mouse tumor cells. *Cancer Treat. Rep.*, **67**, 859.

DANO, K. (1972). Cross resistance between vinca alkaloids and anthracyclines in Ehrlich oxites tumor in vivo. *Cancer Chemoth. Rep.*, **56**, 701.

DANO, K. (1973). Active outward transport of daunomycin in resistant Ehrlich oxites tumor cells. *Biochim. Biophys. Acta*, **323**, 466.

ELLIOT, E.M. & LING, V. (1981). Selection and characterization of Chinese hamster ovary cell mutants resistant to melphalan (L-phenylanaline mustard). *Cancer Res.*, **41**, 393.

GIAVAZZI, R., SCHOLAR, E. & HART, I.R. (1983). Isolation and preliminary characterization of an adriamycin-resistant murine fibrosarcoma cell line. *Cancer Res.*, **43**, 2216.

HAMILTON, T.C., FOSTER, B.J., GROTZINGER, K.R., McKOY, W.M., YOUNG, R.C. & OZOLS, R.F. (1983). Development of drug sensitive and resistant human ovarian cancer cell lines. *Proc. Amer. Assoc. Cancer Res.*, **24**, 313.

HILL, B.T., DENNIS, L.Y., LI, X.T. & WHELAN, R.D.H. (1985). Identification of anthracycline analogues with enhanced cytoxicity and lack of cross-resistance to adriamycin using a series of mammalian cell lines in vitro. *Cancer Chemoth. Pharmacol.*, **14**, 194.

INABA, M., KOBAYASUI, H., SAKURAI, Y. & JOHNSON, R.K. (1979). Active efflux of daunorubicin and adriamycin in sensitive and resistant sublines of P388 leukaemia. *Cancer Res.*, **39**, 2200.

JOHNSON, R.K., OVEJERA, A.A. & GOLDIN, A. (1976). Activity of anthracyclines against an adriamycin-resistant subline of P388 leukaemia with special emphasis on cinerubin A. *Cancer Treat. Rep.*, **60**, 99.

KARTNER, N., RIORDAN, J.R. & LING, V. (1983). Cell surface P-glycoprotein associated with multidrug resistance in mammalian cell lines. *Science*, **221**, 1285.

KESSEL, D., BOTTERILL, V. & WODINSKY, I. (1968). Uptake and retention of daunomycin by mouse leukaemic cells as factors in drug response. *Cancer Res.*, **28**, 938.

LING, V. (1982). Genetic basis of drug resistance in mammalian cells. In *Drug and Hormone Resistance in Neoplasia*, Bruchovsky, N. & Goldie, J.H. (eds.) p 1. CRC Press: Boca Raton.

MUNGIKAR, A., CHITNIS, M. & GOTHOSKAR, B. (1981). Mixed-function oxidase enzymes in adriamycin-sensitive and resistant sublines of P388 leukaemia. *Chem. Biol. Interact.*, **35**, 119.

NISHIMURA, T., MUTO, K. & TANAKA, N. (1978). Drug sensitivity of an adriamycin-resistant mutant subline of mouse lymphoblastoma L5178Y cells. *J. Antibiot. (Tokyo)*, **31**, 493.

RONINSON, I.B., ABELSON, H.T., HOUSMAN, D.E., HOWELL, N. & VARSHAVSKY, A. (1984). Amplification of specific DNA sequences correlates with multi-drug resistance in Chinese hamster cells. *Nature*, **309**, 626.

SCHABEL, F.M., SKIPPER, H.E., TRODER, M.W., LASTER, W.R., GRISWOLD, D.P. & CORBETT, T.H. (1983). Establishment of cross-resistance profiles for new agents. *Cancer Treat. Rep.*, **67**, 905.

SCHWARTZ, H.S. (1973). Fluorometric assay for daunomycin and adriamycin in animal tissues. *Biomed. Med.*, **7**, 396.

SCOTT, C.A., WESTMACOTT, D., BROADHURST, M.J., THOMAS, G.J. & HALL, M.J. (1985). 9-alkyl anthracyclines. Absence of cross-resistance in a human cell line. *Br. J. Cancer*, **52**, 423 (*abstract*).

SKOVSGAARD, T. (1978). Mechanisms of resistance to daunorubicin by sensitive and anthracycline-resistant sublines of P388 leukaemia. *Biochem. Pharmacol.*, **27**, 2123.

TSURUO, T., IIDA, H., TSUKAGOSHI, S. & SAKURAI, Y. (1982). Increased accumulation of vincristine and adriamycin in drug-resistant P388 tumor cells following incubation with calcium antagonists and calmodulin inhibitors. *Cancer Res.*, **42**, 4730.

TSURUO, T., IIDA, H., TSUKAGOSHI, S. & SAKURAI, Y. (1983*a*). Potentiation of vincristine and adriamycin effects in human haemopoietic tumor cell lines by calcium antagonists and calmodulin inhibitors. *Cancer Res.*, **43**, 2267.

TSURUO, T., IIDA, H., TSUKAGOSHI, S. & SAKURAI, Y. (1983*b*). Circumvention of vincristine and adriamycin resistance in vitro and in vivo by calcium influx blockers. *Cancer Res.*, **43**, 2905.

TWENTYMAN, P.R., FOX, N.E., WRIGHT, K.A., WORKMAN, P., BROADHURST, M.J. & MARTIN, J.A. (1985). New anthracyclines. In vitro effects and cross resistance patterns in control and adriamycin-resistant cell lines. *Br. J. Cancer*, **52**, 423 (*abstract*).